# Aquacobalamin Accelerates Orange II Destruction by Peroxymonosulfate via the Transient Formation of Secocorrinoid: A Mechanistic Study

**DOI:** 10.3390/ijms231911907

**Published:** 2022-10-07

**Authors:** Ilia A. Dereven’kov, Ekaterina S. Sakharova, Vladimir S. Osokin, Sergei V. Makarov

**Affiliations:** Department of Food Chemistry, Ivanovo State University of Chemistry and Technology, Sheremetevskiy Str. 7, 153000 Ivanovo, Russia

**Keywords:** peroxymonosulfate, vitamin B_12_, aquacobalamin, azo-dyes, oxidation

## Abstract

Besides its use in medicine, vitamin B_12_ (cobalamin) and its derivatives have found in numerous applications as catalysts. However, studies related to the activation of oxidants via cobalamin are scant. In this work, we showed how the addition of aquacobalamin (H_2_OCbl) accelerates the destruction of azo-dye Orange II by peroxymonosulfate (HSO_5_^−^) in aqueous solutions. In neutral and weakly alkaline media, the process is initiated by the modification of the corrin macrocycle with HSO_5_^−^, which requires the preliminary deprotonation of the aqua-ligand in H_2_OCbl to give hydroxocobalamin, producing 5,6-dioxo-5,6-secocobalamin or its isomer (14,15-dioxo-14,15-secocobalamin). In acidic solutions, where the concentration of hydroxocobalamin is negligible, the formation of dioxo-seco-species is not observed, and the reaction between H_2_OCbl and HSO_5_^−^ results in slow chromophore bleaching. Using terephthalic acid, we demonstrated the formation of hydroxyl radicals in the mixture of H_2_OCbl with HSO_5_^−^, whereas the generation of sulfate radicals was proved by comparing the effects of ethanol and nitrobenzene on Orange II destruction using the H_2_OCbl/HSO_5_^−^ system. The reaction mechanism includes the binding of HSO_5_^−^ to the Co(III) ion of dioxo-secocobalamin, which results in its deprotonation and the labilization of the O-O bond, leading to the formation of sulfate and hydroxyl radicals which further react with Orange II.

## 1. Introduction

Peroxymonosulfate (HSO_5_^−^) is a frequently used ion in advanced oxidation processes due to its ability to generate sulfate radicals (SO_4_^•−^) [1,2]. SO_4_^•−^ exhibits extremely high oxidizing properties [3], i.e., the oxidation potential is 2.5–3.1 V (vs. a normal hydrogen electrode, NHE) [4], and is capable of reacting with numerous organic and inorganic molecules [3,5]. Cobalt compounds efficiently activate the O-O bond in HSO_5_^−^ [6]. It was suggested that cobalt species act as a Fenton reagent in the reaction with HSO_5_^−^ [6]. However, the most recent explanation of the mechanism of Co(II)-assisted HSO_5_^−^ activation includes the consequent binding of two SO_5_^2−^ molecules which results in O-O bond labilization in one of SO_5_^2−^ ligands, and the liberation of sulfate radicals [7]. Another study demonstrates the pronounced oxidizing properties of the Co(II)-SO_5_^2−^ complex as a primary intermediate in the Co(II)/peroxymonosulfate system [8]. Cobalt tetrapyrrolic complexes have been used in the activation of peroxymonosulfate as well. For example, cobalt phthalocyanine immobilized onto cellulose fiber demonstrated high efficiency in the decolorization of azo-dyes by HSO_5_^−^, which increased upon the addition of bicarbonate. The catalytic cycle included the coordination of SO_5_^2−^ with Co(II) and the further formation of high-valent oxo-species [9]. Another study employed molecular sieves containing cobalt tetracarboxyl phthalocyanine and manganese ions for diclofenac destruction via HSO_5_^−^. The process involved the generation of singlet oxygen as a major reactive oxidant as well as sulfate and hydroxyl radicals [10].

**Figure 1:**
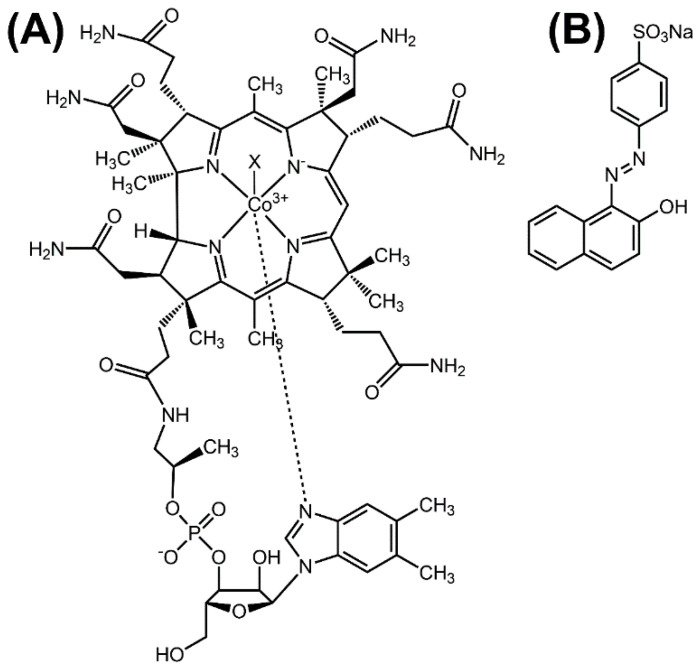
Structures of cobalamin (**A**; X = H_2_O; CN^−^ and others) and Orange II (**B**).

**Figure 2:**
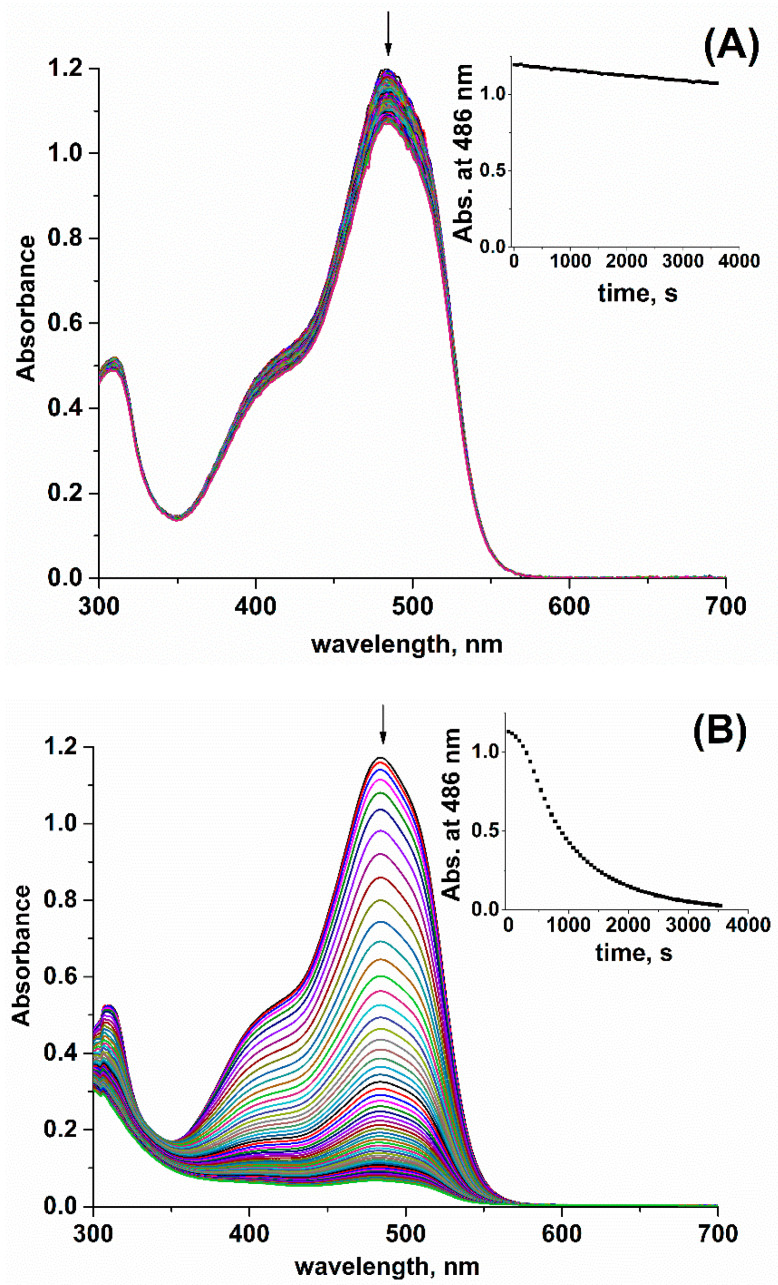
(**A**) UV-vis spectra of the reaction between Orange II (5.7·10^−5^ M) and HSO_5_^−^ (5.0·10^−4^ M) at pH 7.4, 25.0 °C. (**B**) UV-vis spectra of the reaction between Orange II (5.7·10^−5^ M) and HSO_5_^−^ (5.0·10^−4^ M) in the presence of H_2_OCbl (1.0·10^−6^ M) at pH 7.4, 25.0 °C. The time interval between the spectra is 60 s. The total reaction time is 60 min. Inset: time-course curves of the reactions.

Cobalamins (Cbls; Figure 1A) are the most ubiquitous cobalt complexes in nature. The catalytic behavior of Cbls has been characterized in numerous systems [11,12,13]. However, the application of corrinoids in the activation of oxidants found limited attention. For example, heptamethyl cobyrinate catalyzes the oxidation of alkanes to their corresponding alcohols and ketones by m-chloroperbenzoic acid via the transient formation of the acylperoxido complex [14]. A complex with hydrogen peroxide has been reported for the Co(III) form of Cbl (Cbl(III)) [15], whereas the reaction of the Co(II) form of Cbl (Cbl(II)) with hydrogen peroxide leads to corrin ring modification [16,17]. Cyanocobalamin (CNCbl) was successfully used as an electrocatalyst in water oxidation [18]. The computational work suggests a relatively complex mechanism in the process, in which Cbl acts as a redox non-innocent complex [19]. CNCbl was used in the synthesis of a cobalt-containing composite, which was employed in HSO_5_^−^ activation. However, CNCbl was subjected to pyrolysis, which resulted in the destruction of its structure [20]. In this work, we report that the addition of H_2_OCbl accelerates the oxidation of azo-dye Orange II (Figure 2B) by HSO_5_^−^ in aqueous solutions, and we provide the mechanistic details of this process. Orange II has been used earlier as a model compound in other systems, including cobalt derivatives and HSO_5_^−^ as well [21,22,23,24].

## 2. Results and Discussion

Orange II bleaching by HSO_5_^−^ proceeds slowly in a neutral medium in the absence of H_2_OCbl (Figure 2A). However, the addition of H_2_OCbl accelerates Orange II destruction by HSO_5_^−^ accompanied by a decrease in the absorbance in the range between 300 and 600 nm (Figure 2B). Note that H_2_OCbl (1.0·10^−6^ M) and HSO_5_^−^ (5.0·10^−4^ M) weakly absorb in the UV-vis spectrum in comparison with Orange II (Supplementary Figure S1). Kinetic curves of the reaction have a sigmoid profile that can be explained by the transformation of H_2_OCbl into other complexes possessing catalytic activity.

**Figure 3:**
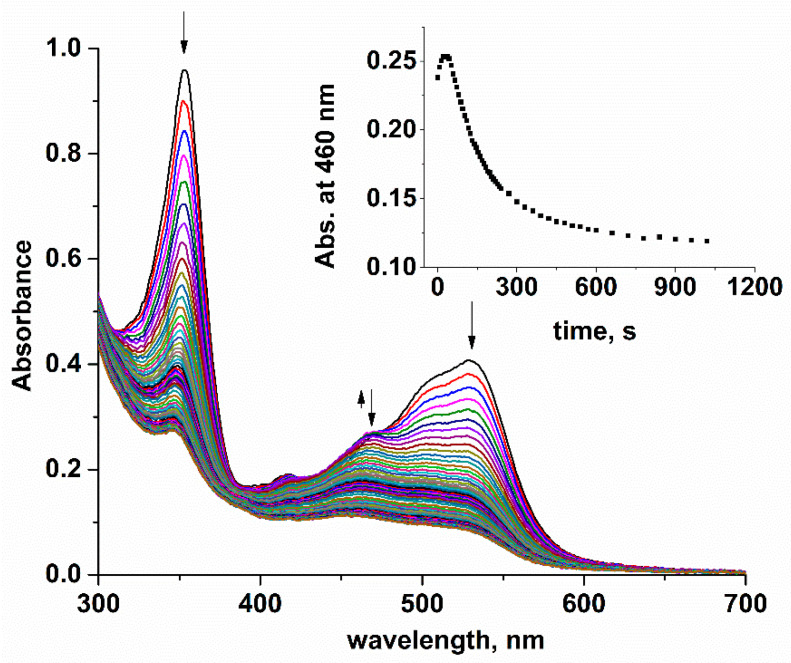
UV-vis spectra of the reaction between H_2_OCbl (5.0·10^−5^ M) and HSO_5_^−^ (1.0·10^−3^ M) at pH 7.4, 25.0 °C. Time intervals between the spectra are 10, 30, and 60 s for 0–4, 4.5–10 and 10–17 min of the reaction, respectively. Maxima at 353, 505, and 528 nm correspond to H_2_OCbl, and maximum at ca. 470 nm—the product of H_2_OCbl modification by HSO_5_^−^ (dioxo-secocorrinoid). Inset: a time-course curve of the reaction.

To identify catalyst species formed in the mixture of H_2_OCbl with HSO_5_^−^ and elucidate the mechanistic details of Orange II destruction in this system, we studied the reaction between H_2_OCbl and HSO_5_^−^. Since H_2_OCbl exhibits very weak absorbance in the micromolar concentration range, which was employed in most of the experiments in this study (Supplementary Figure S1), higher H_2_OCbl concentrations were used to control this process. Figure 3 shows UV-vis spectra of the reaction between H_2_OCbl and the excess of HSO_5_^−^ at pH 7.4, i.e., the formation of species with a maximum at ca. 470 nm is observed at the beginning of the reaction, when the destruction of chromophore occurs. UV-vis spectra recorded after the incubation of H_2_OCbl with different concentrations of HSO_5_^−^ indicate that H_2_OCbl cannot be completely converted into the species absorbing at ca. 470 nm, which can be explained by their low stability in the presence of HSO_5_^−^ (Supplementary Figure S2).

**Figure 4:**
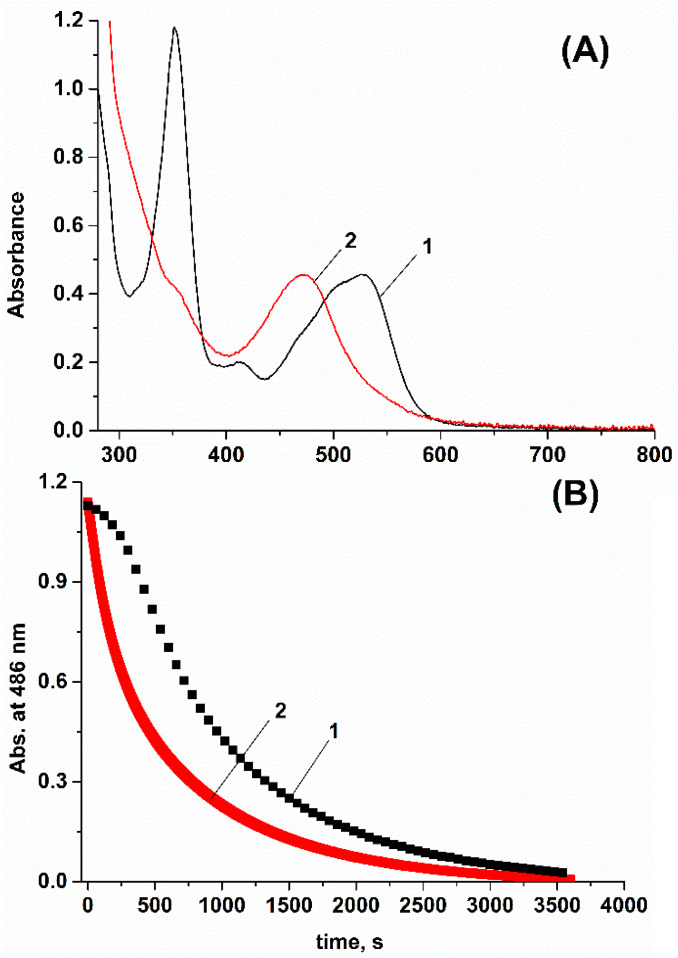
(**A**) UV-vis spectra of H_2_OCbl (5.0·10^−5^ M; spectrum 1) and the products generated by the reaction between H_2_OCbl and the two-fold excess of HSO_5_^−^ (spectrum 2), recorded at pH 7.4, 25.0 °C. (**B**) Time-course curves of the Orange II (5.8·10^−5^ M) destruction in the mixtures of HSO_5_^−^ (5.0·10^−4^ M) with H_2_OCbl (1.0·10^−6^ M; curve 1) and the products generated by the reaction between H_2_OCbl and the two-fold excess of HSO_5_^−^ (ca. 1.0·10^−6^ M; curve 2) at pH 7.4, 25.0 °C.

**Scheme 1:**
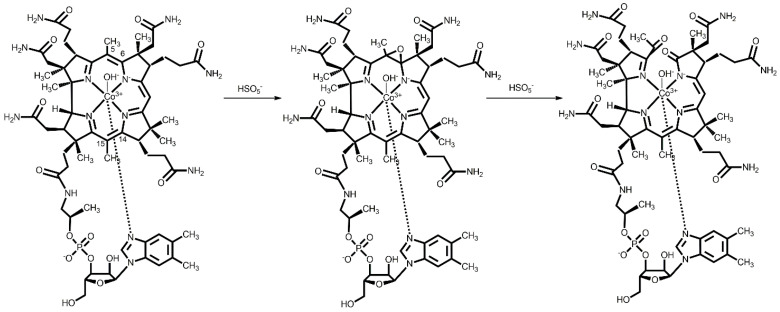
Mechanism of hydroxocobalamin modification by HSO_5_^−^. Similar parallel reactions proceed with a double bond between the C14 and C15 atoms of the corrin ring as well.

The products of the reaction between H_2_OCbl and the two-fold excess of HSO_5_^−^ were separated from unreacted H_2_OCbl using column chromatography. UV-vis spectrum of products is shown in Figure 4. It includes a maximum at 472 nm and lacks a γ-band (maximum at 300–400 nm). The same observations have been reported earlier for the reaction involving dicyanocobester and singlet oxygen photogenerated in an aerobic methanolic solution containing methylene blue, which produces a mixture of 5,6-dioxo-5,6-seco- and 14,15-dioxo-14,15-secocorrinoids (complexes with a cleaved corrin ring with a structure of 5,6-dioxo-5,6-secocobalamin are presented in Scheme 1) [25]. The formation of 5,6-dioxo-5,6-seco- or 14,15-dioxo-14,15-secocorrinoids in the course of the reaction between H_2_OCbl and HSO_5_^−^ is supported by MALDI-mass-spectroscopy as well. The mass-spectrum of the products includes a major peak at *m*/*z* = 1383.5 (Supplementary Figure S3), which can be attributed to the [Cbl(II) – H + Na + 2O]^+^ ion corresponding to dioxo-seco-Cbl. A minor peak in the mass-spectrum at *m*/*z* = 1399.5 can be assigned to hydroxylated dioxo-seco-Cbl, i.e., a product of the further reaction between dioxo-seco-Cbl and HSO_5_^−^. 

The maximum in the UV-vis spectrum of H_2_OCbl modification by HSO_5_^−^ at 472 nm can be erroneously ascribed to yellow corrinoids, i.e., corrinoid derivatives hydroxylated at the C5- or C15-position of the corrin ring and lacking double bonds between the C4-C5 and C5-C6 or C14-C15 and C15-C16 atoms. However, UV-vis spectra of yellow corrinoids exhibit a γ-band, which is slightly less intense in comparison with the band of unmodified corrinoids [26,27]. 

The kinetic curves of Orange II bleaching in mixtures containing HSO_5_^−^ and H_2_OCbl or its dioxo-seco derivatives are shown in Figure 4. In the case of the dioxo-seco derivatives of H_2_OCbl, the Orange II destruction proceeds faster and kinetic curves do not include the induction period that supports the involvement of dioxo-seco derivatives in Orange II destruction by HSO_5_^−^. 

The intensity of the absorption maximum in the UV-vis spectrum is characteristic of dioxo-secocorrinoids (472 nm), emerging upon H_2_OCbl mixing with HSO_5_^−^, depending on the pH. At pH 4.5, this peak is negligible (Supplementary Figure S4), and slow chromophore bleaching occurs. The peak at 472 nm becomes more pronounced in a neutral medium (Figure 3) and reaches the highest intensity at pH 9.2 (Supplementary Figure S5). This observation can be explained by the transformation of H_2_OCbl to hydroxocobalamin (pK_a_(H_2_OCbl) = 7.8 at 25.0 °C [28]), which is capable of reacting with HSO_5_^−^ to give dioxo-secocorrinoids. In an acidic medium, Cbl exists in an aqua-form, which reacts with HSO_5_^−^ via chromophore degradation. In comparison with water molecules, hydroxide possesses more pronounced nucleophilic properties and likely increases the electron density of the macrocycle, which facilitates its modification with HSO_5_^−^. The effect of the upper-axial ligands of Cbls on the structure and yield of corrin-modified species has been reported in earlier work [29]. 

The reaction between H_2_OCbl and HSO_5_^−^ is almost unaffected by the addition of ethanol, which acts as a scavenger of hydroxyl [30,31] and sulfate [32] radicals generated upon O-O bond homolysis in HSO_5_^−^ (Supplementary Figure S6). The activation of HSO_5_^−^ can result in the formation of singlet oxygen [33,34] reacting with H_2_OCbl to give dioxo-secocorrinoinds [26]. However, the addition of tryptophan, an efficient quencher of singlet oxygen [35], does not affect the reaction between H_2_OCbl and HSO_5_^−^ (Supplementary Figure S7), i.e., participation of singlet dioxygen in the process is unlikely. Therefore, hydroxocobalamin is modified by HSO_5_^−^ but not by its decomposition products. Obviously, hydroxocobalamin subsequently reacts with two HSO_5_^−^ molecules via the epoxidation of C5-C6 or C14-C15 bonds and their further cleavage (Scheme 1).

**Figure 5:**
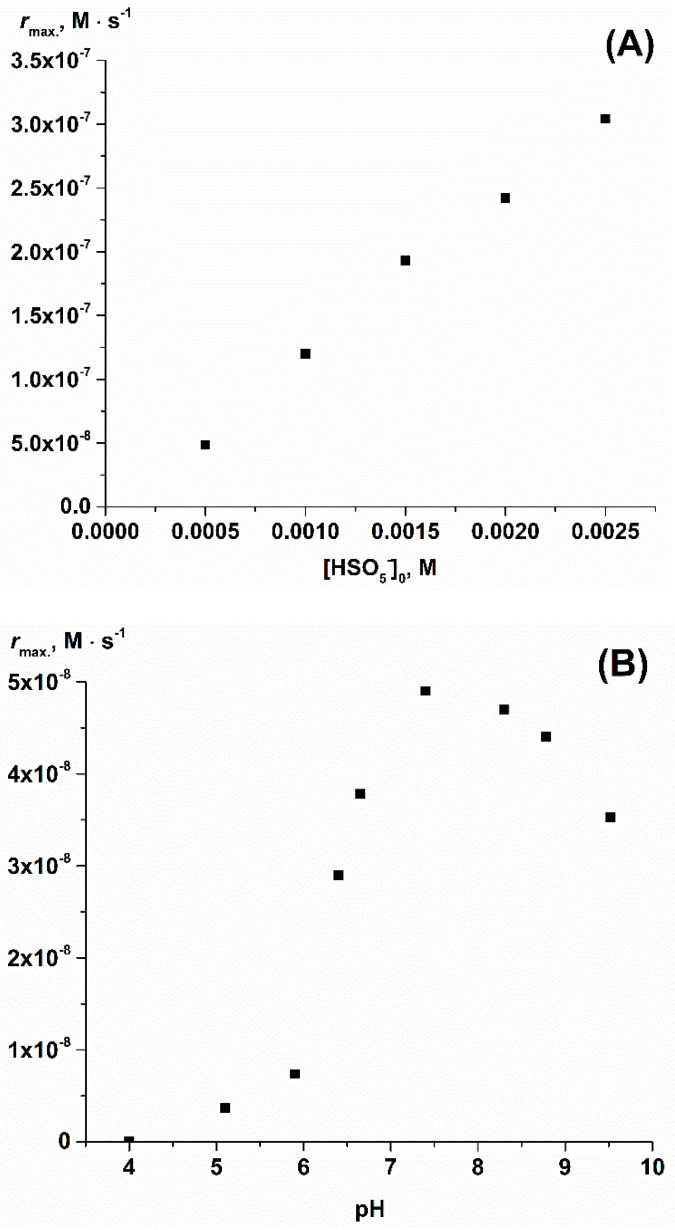
(**A**) The plot of the maximum rate of the reaction between Orange II (5.7·10^−5^ M) and HSO_5_^−^ in the presence of H_2_OCbl (1.0·10^−6^ M) versus the initial concentration of HSO_5_^−^ at pH 7.4, 25.0 °C. (**B**) The plot of the maximum rate of the reaction between Orange II (5.7·10^−5^ M) and HSO_5_^−^ (5.0·10^−4^ M) in the presence of H_2_OCbl (1.0·10^−6^ M) versus pH at 25.0 °C.

Next, we studied the kinetics of Orange II destruction in the presence of HSO_5_^−^ and H_2_OCbl. The dependence of the maximum rate of the reaction on the initial H_2_OCbl concentration is shown in Supplementary Figure S8. It is non-linear and reaches a plateau at [H_2_OCbl] > 2.0·10^−5^ M. This observation can be explained by the decomposition of catalyst species and by HSO_5_^−^ disproportionation that becomes more pronounced in the presence of high H_2_OCbl concentrations. The maximum rate of Orange II destruction by HSO_5_^−^ in the presence of H_2_OCbl linearly depends on the HSO_5_^−^ concentration (Figure 5A). This implies that the catalyst reacts with one HSO_5_^−^ molecule upon the generation of an oxidant reacting with the dye. The dependence of the maximum rate of Orange II oxidation by HSO_5_^−^ in the presence of H_2_OCbl on pH is shown in Figure 5B. It exhibits a bell-shaped profile with a maximum at ca. pH 8 that results from the influence of two acid-base equilibria on the reaction kinetics. One of these equilibria includes the formation of hydroxocobalamin (p*K*_a_(H_2_OCbl) = 7.8 at 25.0 °C [28]) that facilitates the formation of dioxo-secocobalamins upon an increase in pH. The second one is a deprotonation of HSO_5_^−^ (p*K*_a_ = 9.3 at 25.0 °C [34]) that decreases its stability [1]. 

Using low concentrations of HSO_5_^−^ to prevent the rapid formation of dioxo-secocorrinoids, we determined the initial rates of Orange II bleaching mediated by H_2_OCbl, but not by the products of its decomposition. The dependence of the initial rate on HSO_5_^−^ is linear (Supplementary Figure S9) with the slope (2.2 ± 0.2)·10^−5^ s^−1^ (pH 7.4, 25.0 °C). For the dependence presented in Figure 5A, which predominantly reflects the reaction mediated by dioxo-secocorrinoids, the slope is (1.3 ± 0.1)·10^−4^ s^−1^ (pH 7.4, 25.0 °C). Thus, the reaction mediated by dioxo-secocobalamin is ca. 10-fold more efficient than by H_2_OCbl.

**Figure 6:**
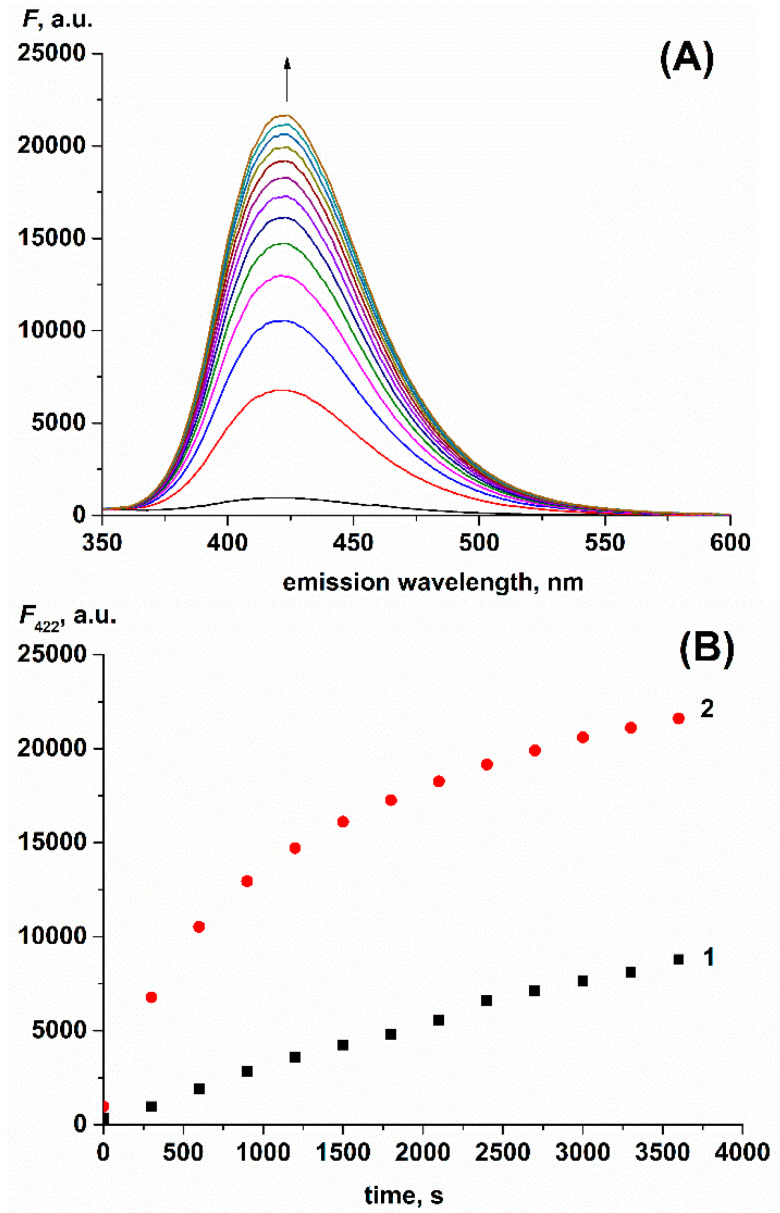
Fluorescence emission spectra of the mixture of terephthalic acid (1.0·10^−3^ M) with H_2_OCbl (1.0·10^−6^ M) and HSO_5_^−^ (5.0·10^−4^ M) were recorded every 5 min after mixing (**A**), and plots of fluorescence intensity at 422 nm versus time for mixtures of terephthalic acid (1.0·10^−3^ M) with HSO_5_^−^ (5.0·10^−4^ M; Line 1) and with HSO_5_^−^ (5.0·10^−4^ M) and H_2_OCbl (1.0·10^−6^ M; Line 2; **B**) at pH 7.4, 25.0 °C.

We attempted to identify those species formed from HSO_5_^−^ that are responsible for the reaction with Orange II in the course of activation via dioxo-secocobalamins. The formation of hydroxyl radicals can be monitored using terephthalic acid, which produces, during this process, highly fluorescent 2-hydroxyterephthalic acid (Supplementary Figure S10) [36,37]. Indeed, 2-hydroxyterephthalic acid is generated in the mixture of terephthalic acid with HSO_5_^−^ in the absence or in the presence of H_2_Ocbl. However, the addition of H_2_OCbl noticeably accelerates its formation (Figure 6). Alternatively, the formation of 2-hydroxyterephthalic acid can be suggested via a route involving the generation of singlet oxygen from the HSO_5_^−^ peroxidation of terephthalic acid and the decomposition of the peroxides. To elucidate the type of species hydroxylating terephthalic acid, we examined the effect of ethanol on 2-hydroxyterephthalic acid formation in the abovementioned systems. Ethanol does not react with singlet oxygen in contrast to the hydroxyl radical [30,31]. We found that the addition of ethanol significantly decreases the fluorescence intensity of the 2-hydroxyterephthalic acid generated from terephthalic acid and HSO_5_^−^ or HSO_5_^−^/H_2_OCbl systems (Supplementary Figure S11), which supports the formation of the hydroxyl radical.

**Figure 7:**
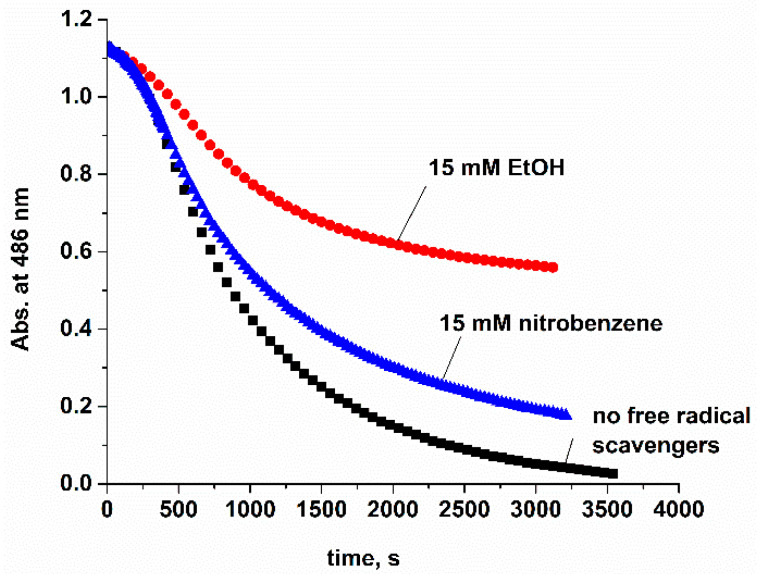
Time-course curves for Orange II (5.5·10^−5^ M) destruction via the mixture of H_2_OCbl (1.0·10^−6^ M) with HSO_5_^−^ (5.0·10^−4^ M) at pH 7.4, 25.0 °C in the absence or in the presence of ethanol (1.5·10^−2^ M) or nitrobenzene (1.5·10^−2^ M).

We compared the effect of equal concentrations of ethanol and nitrobenzene on Orange II bleaching in the presence of H_2_OCbl and HSO_5_^−^ since hydroxyl and sulfate radicals possess comparable reactivity toward ethanol, whereas the sulfate radical is less reactive toward nitrobenzene [38] than to the hydroxyl radical [39,40]. Figure 7 indicates that inhibition of the reaction is more pronounced in the case of ethanol than in the presence of nitrobenzene. This result suggests the generation of the sulfate radical upon HSO_5_^−^ activation via the dioxo-secocobalamins.

The phosphate ions used to maintain pH in the course of the experiments in this work may affect reactions involving peroxymonosulfate [41]. However, phosphate buffer concentration weakly affects the kinetics of Orange II bleaching by the H_2_OCbl/HSO_5_^−^ system (Supplementary Figure S12). A more significant effect was observed in the case of the bicarbonate ion, i.e., the addition of HCO_3_^−^ substantially decreases the rate of Orange II destruction via the mixture of HSO_5_^−^ with H_2_OCbl in a neutral medium (Supplementary Figure S13), which can be explained by the rapid scavenging of HO^•^ [40,42] and SO_4_^•−^ [43] by HCO_3_^−^ to give a carbonate radical less reactive toward Orange II. 

**Scheme 2:**
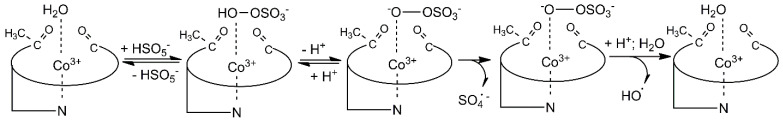
Mechanism of HSO_5_^−^ activation by dioxo-secocobalamins.

Thus, HO^•^ and SO_4_^•−^ are formed upon HSO_5_^−^ activation via dioxo-secocobalamins. Probably, the binding of HSO_5_^−^ via the Co(III) ion of dioxo-secocobalamins facilitates its deprotonation and labilization of the O-O bond (Scheme 2). It is well known that the Co(III) ion in Cbls exhibits a relatively soft metal center [26,44], whereas corrin ring cleavage to give the dioxo-seco species makes the Co(III) ion harder [45]. Therefore, the coordination of HSO_5_^−^, a hard base, is more plausible on the Co(III) ion in dioxo-seco-Cbl than in unmodified Cbl. 

In contrast to H_2_OCbl, CNCbl weakly affects the rate of Orange II destruction in the presence of HSO_5_^−^ (Supplementary Figure S14). The UV-vis spectra indicate that the modification of CNCbl occurs via HSO_5_^−^ (Supplementary Figure S15). However, no new maxima at 470–500 nm, typical to dioxo-secocorrinoids, were observed. Moreover, cyanide remains tightly bound with the Co(III) ion upon corrin modification [46] and prevents the reaction of HSO_5_^−^ with Co(III) to generate species that react with Orange II. 

Thus, this work showed that the destruction of azo dye Orange II is accelerated after the addition of aquacobalamin. Aquacobalamin retains its catalytic properties even after partial destruction. Moreover, an increase in the catalytic properties of H_2_OCbl is observed upon its partial destruction, i.e., dioxo-secocorrionoids can be more efficient oxidation catalysts in the activation of the peroxo species. Thus, the elaboration of the catalytic effect of modified corrinoids is the prospective topic for further studies.

## 3. Materials and Methods

Hydroxocobalamin hydrochloride (Sigma, St. Louis, MO, USA; HOCbl; ≥96%), Oxone (Sigma; 2KHSO_5_ · K_2_SO_4_ · KHSO_4_), terephthalic acid (TA; Sigma-Aldrich, St. Louis, MO, USA; 98%), 2-hydroxyterephthalic acid (J&K), Orange II sodium salt (Sigma-Aldrich; ≥85%) were used without additional purification. The content of KHSO_5_ in OXONE was determined by the reported procedure [7]. Concentrations of Cbl stock solutions were determined using UV-visible spectroscopy via conversion of Cbl to its dicyano-form (extinction coefficient is 30,400 M^−1^∙cm^−1^ at 368 nm [47]).

Buffer solutions (phosphate or its mixture with acetate or tetraborate; 0.1 M) were used to maintain pH during the measurements. The pH values of the solutions were determined using Multitest IPL-103 pH-meter (SEMICO) equipped with an ESK-10601/7 electrode (Izmeritelnaya tekhnika) filled with 3.0 M KCl solution. The electrode was preliminarily calibrated using standard buffer solutions (pH 1.65–12.45). 

Ultraviolet-visible (UV–vis) spectra were recorded on a cryothermostated (±0.1 °C) Shimadzu UV-1800 and Cary 50 UV–Vis spectrophotometers in quartz cells.

Fluorescence emission spectra were recorded on a Shimadzu RF-6000 spectrofluorophotometer. The excitation wavelength was 315 nm, and the excitation and emission bandwidths were 1.5 and 20.0 nm, respectively.

Separation of products of the reaction between H_2_OCbl and the two-fold excess of HSO_5_^−^ at pH 7.4 from unreacted H_2_OCbl was performed using column chromatography on silica gel (Sigma-Aldrich; average pore size 60 Å (52–73 Å), 70–230 mesh, 63–200 μm) using 5% aqueous acetic acid as eluent.

MALDI-MS measurements were performed on a Shimadzu AXIMA Confidence mass-spectrometer with 2,5-dihydroxybenzoic acid as the matrix.

## 4. Conclusions

This work demonstrated that the bleaching of azo-dye Orange II by HSO_5_^−^ is accelerated upon the addition of aquacobalamin. The reaction between hydroxocobalamin and HSO_5_^−^ results in corrin ring cleavage and the formation of 5,6-dioxo-5,6-secocobalamin, which participates in the activation of HSO_5_^−^. The mixing together of aquacobalamin with HSO_5_^−^ and terephthalic acid generates 2-hydroxyterephthalic acid more efficiently than in the absence of H_2_Ocbl, indicating the formation of hydroxyl radicals. In the presence of ethanol, which acts as an efficient scavenger of hydroxyl and sulfate radicals, the bleaching of the Orange II by the H_2_OCbl/HSO_5_^−^ mixture proceeds less efficiently, whereas, with the effect of nitrobenzene, which is less reactive toward SO_4_^•−^ than it is toward HO^•^, the inhibition of the reaction was less pronounced. These results confirm the important role of SO_4_^•−^ in the destruction of Orange II. The strong inhibition of Orange II bleaching was observed upon adding bicarbonate to the H_2_OCbl/HSO_5_^−^ system; this can be explained by the reaction of HCO_3_^−^ with SO_4_^•−^ and HO^•^ to give a less reactive carbonate radical. The suggested mechanism of HSO_5_^−^ activation by dioxo-secocobalamin includes the formation of a complex between the Co(III) ion and HSO_5_^−^ which leads to peroxymonosulfate deprotonation and the labilization of the O-O bond, also resulting in the formation of the hydroxyl and sulfate radicals. In contrast to H_2_OCbl, cyanocobalamin weakly affects the rate of Orange II bleaching by HSO_5_^−^, which can be explained by the absence of dioxo-secocorrinoid formation upon the reaction of cyanocobalamin with HSO_5_^−^, as well as by the presence of cyanide bound to cobalt in cobalamin-derived species, preventing the reaction between the cobalt ions and HSO_5_^−^.

## Data Availability

Not applicable.

## Supplementary Materials

The following supporting information can be downloaded at: https://www.mdpi.com/article/10.3390/ijms231911907/s1. Figure S1. UV-vis spectra of Orange II (5.7·10^−5^ M), H2OCbl (1.0·10^−6^ M) and HSO^5−^ (5.0·10^−4^ M) at pH 7.4, 25.0 °C; Figure S2. UV-vis spectra of the mixtures of H2OCbl (5.0·10^−5^ M) with different quantities of HSO^5−^ recorded after 5 hours of incubation at pH 7.4, 25.0 °C; Figure S3. MALDI-mass-spectrum of the products of the reaction between H2OCbl and two-fold excess of HSO^5−^; Figure S4. UV-vis spectra of the reaction between H2OCbl (5.0·10^−5^ M) and HSO^5−^ (1.0·10^−3^ M) at pH 4.5, 25.0 °C.; Figure S5. UV-vis spectra of the reaction between H2OCbl (5.0·10^−5^ M) and HSO^5−^ (1.0·10^−3^ M) at pH 9.2, 25.0 °C. Figure S6. UV-vis spectra for the reaction between H2OCbl (5.0·10^−5^ M) with HSO^5−^ (1.0·10^−3^ M) at pH 7.4, 25.0 °C in the presence of ethanol (5.0·10^−2^ M); Figure S7. UV-vis spectra for the reaction between H2OCbl (5.0·10^−5^ M) with HSO^5−^ (1.0·10^−3^ M) at pH 7.4, 25.0 °C in the presence of tryptophan (5.0·10^−3^ M). Figure S8. Plot of the maximum rate of the reaction between Orange II (5.7·10^−5^ M) and HSO^5−^ (5.0·10^−4^ M) in the presence of H2Ocbl versus initial concentration of H2Ocbl at pH 7.4, 25.0 °C; Figure S9. Plot of the initial rate of the reaction between Orange II (5.7·10^−5^ M) and HSO^5−^ in the presence of H2OCbl (1.0·10^−6^ M) versus initial concentration of HSO^5−^ at pH 7.4, 25.0 °C; Figure S10. Fluorescence emission spectrum of 2-hydroxyterephthalic acid (1.0·10^−6^ M) at pH 7.4, 25.0 °C; Figure S11. Plots of fluorescence intensity at 422 nm versus time for mixtures of terephthalic acid (1.0·10^−3^ M) with HSO^5−^ (5.0·10^−4^ M; A) and with HSO^5−^ (5.0·10^−4^ M) and H2OCbl (1.0·10^−6^ M; B) in the absence and in the presence of ethanol (50 mM) at pH 7.4, 25.0 °C; Figure S12. Time-course curves for the destruction of Orange II (5.5·10^−5^ M) by the mixture of H2OCbl (1.0·10^−6^ M) with HSO^5−^ (5.0·10^−4^ M) at pH 7.4, 25.0 °C in the presence of different phosphate buffer concentrations; Figure S13. UV-vis spectra for the destruction of Orange II (5.7·10^−5^ M) by the mixture of H2OCbl (1.0·10^−6^ M) with HSO^5−^ (5.0·10^−4^ M) at pH 7.4, 25.0 °C in the presence of HCO3− (5.0·10^−2^ M); Figure S14. UV-vis spectra of the reaction between Orange II (5.5·10^−5^ M) and HSO5− (5.0·10^−4^ M) in the presence of CNCbl (1.0·10^−6^ M) at pH 7.4, 25.0 °C; Figure S15. UV-vis spectra for the reaction between CNCbl (5.0·10^−5^ M) with HSO^5−^ (5.0·10^−4^ M) at pH 7.0, 25.0 °C.
