# Supplementary material for: Aquacobalamin Accelerates Orange II Destruction by Peroxymonosulfate via the Transient Formation of Secocorrinoid: A Mechanistic Study
Source: Int J Mol Sci. 2022 Oct 7;23(19):11907. doi: 10.3390/ijms231911907 (PMC9569875; doi:10.3390/ijms231911907)
Supplement: Supplementary file 1 [file ijms-23-11907-s001.zip › ijms-1925567-supplementary.pdf]

Supplementary Material

Aquacobalamin accelerates Orange II destruction by peroxymonosulfate via the transient formation of secocorrinoid: a mechanistic study

Ilia A. Dereven'kov, Ekaterina S. Sakharova, Vladimir S. Osokin, and Sergei V. Makarov*

Department of Food Chemistry, Ivanovo State University of Chemistry and Technology, Sheremetevskiy str. 7, 153000 Ivanovo, Russia

* Author to whom correspondence should be addressed.

E-mail address: makarov@isuct.ru (S.V. Makarov)

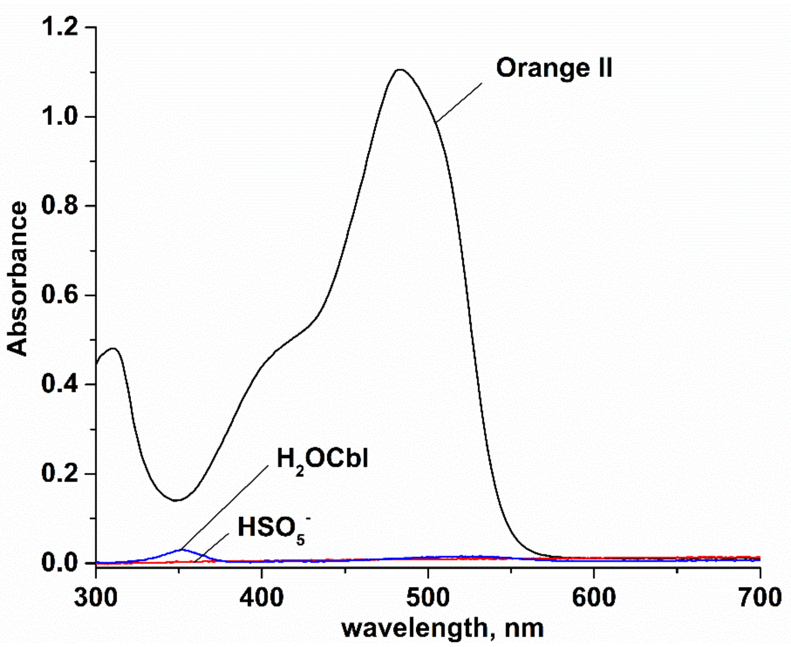

Figure S1. UV-vis spectra of Orange II ($5.7\cdot10^{-5}$ M), H_2OCbl ($1.0\cdot10^{-6}$ M) and HSO_5^- ($5.0\cdot10^{-4}$ M) at pH 7.4, 25.0 °C

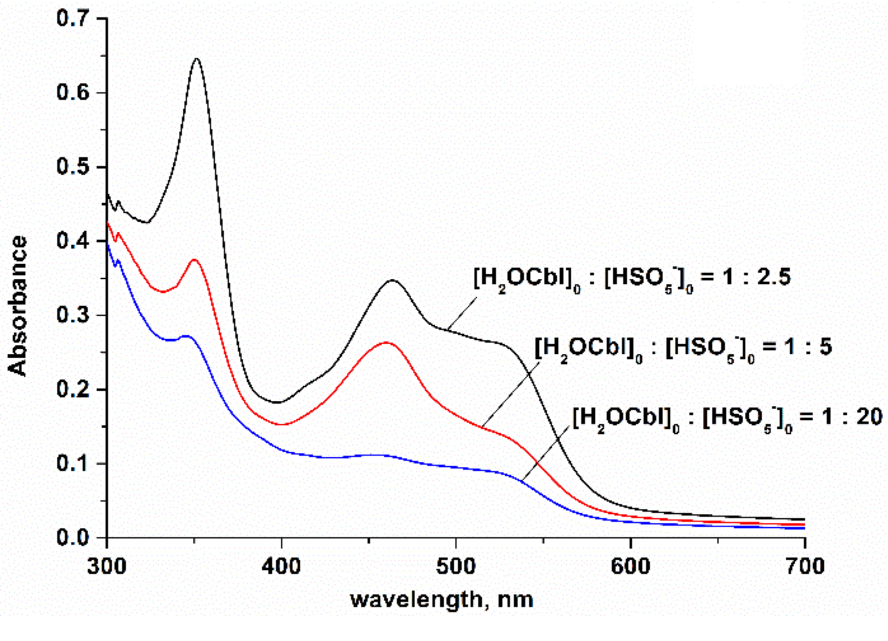

Figure S2. UV-vis spectra of the mixtures of H_2OCbl ($5.0\cdot10^{-5}$ M) with different quantities of HSO_5^- recorded after 5 hours of incubation at pH 7.4, 25.0 °C

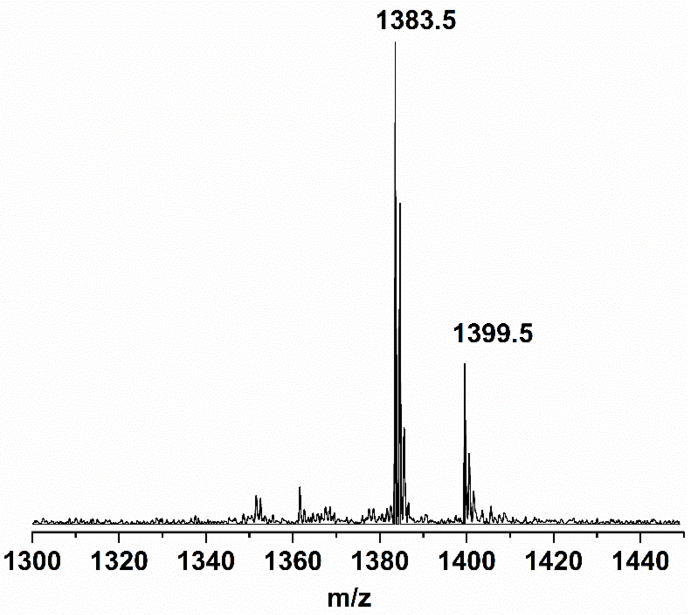

Figure S3. MALDI-mass-spectrum of the products of the reaction between H_2OCbl and two-fold excess of HSO_5^-

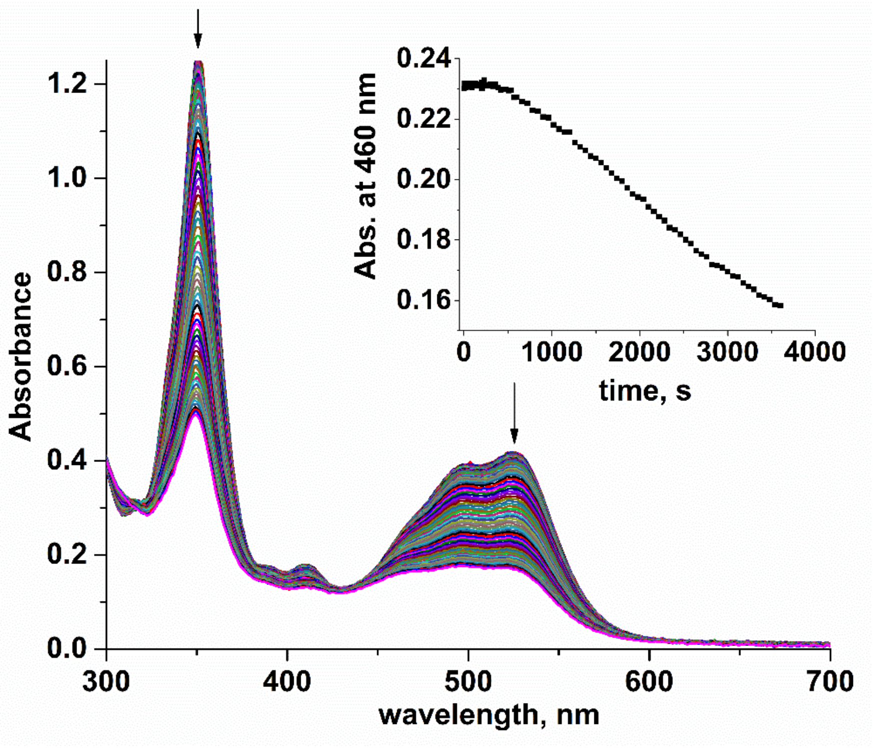

Figure S4. UV-vis spectra of the reaction between H_2OCbl ($5.0 \cdot 10^{-5}$ M) and HSO_5^- ($1.0 \cdot 10^{-3}$ M) at pH 4.5, 25.0 °C. Time intervals between spectra are 10, 30 and 60 s for 0…4, 4.5…10 and 10…60 min of the reaction, respectively. Inset: a time-course curve of the reaction

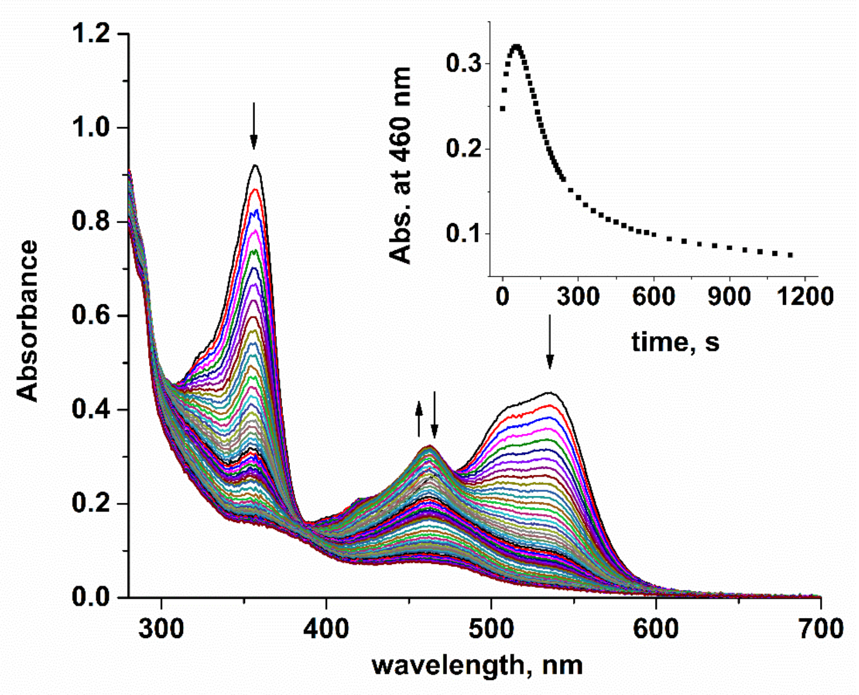

Figure S5. UV-vis spectra of the reaction between H_2OCbl ($5.0 \cdot 10^{-5}$ M) and HSO_5^- ($1.0 \cdot 10^{-3}$ M) at pH 9.2, 25.0 °C. Time intervals between spectra are 10, 30 and 60 s for 0…4, 4.5…10 and 10…19 min of the reaction, respectively. Inset: a time-course curve of the reaction

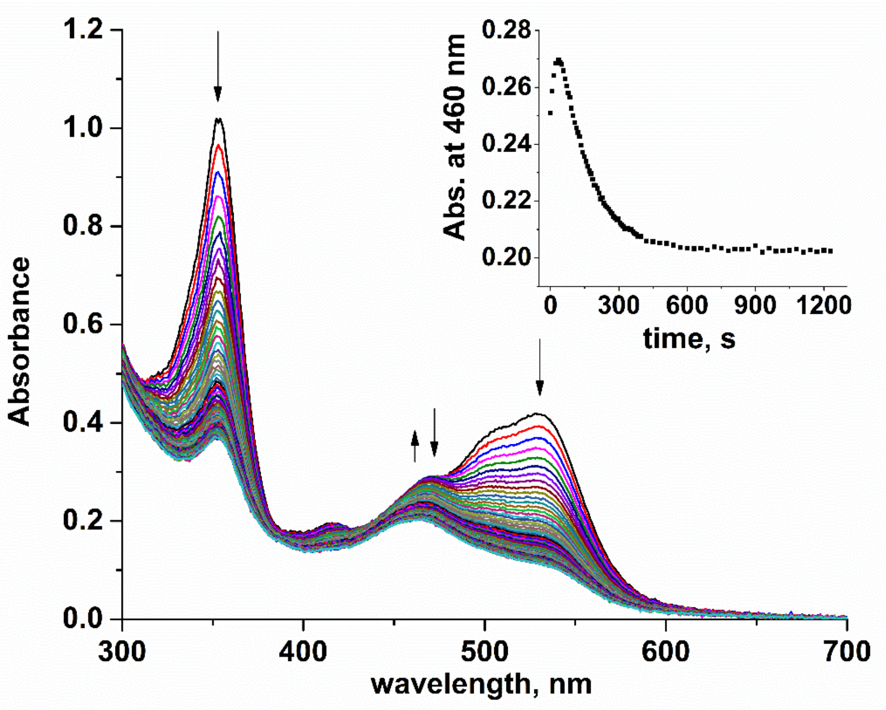

Figure S6. UV-vis spectra for the reaction between H_2OCbl ($5.0 \cdot 10^{-5}$ M) with HSO_5^- ($1.0 \cdot 10^{-3}$ M) at pH 7.4, 25.0 °C in the presence of ethanol ($5.0 \cdot 10^{-2}$ M). Time intervals between spectra are 10 and 30 s for 0…6.5 and 7 …21 min of the reaction, respectively. Inset: a time-course curve of the reaction

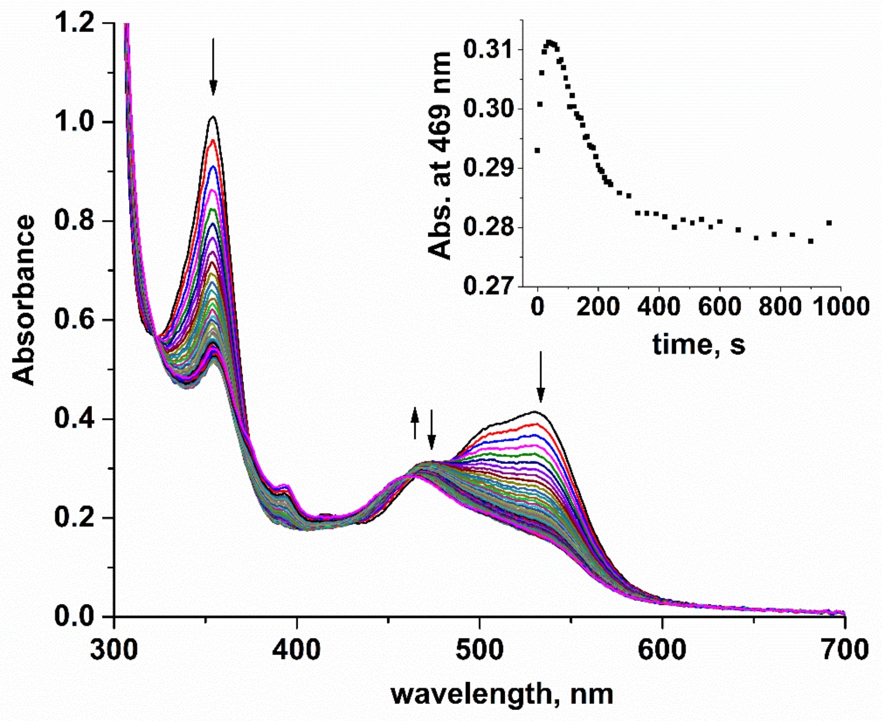

Figure S7. UV-vis spectra for the reaction between H_2OCbl ($5.0\cdot10^{-5}$ M) with HSO_5^- ($1.0\cdot10^{-3}$ M) at pH 7.4, 25.0 °C in the presence of tryptophan ($5.0\cdot10^{-3}$ M). Final spectra of the reaction are contributed by absorbance of tryptophan oxidation products. Time intervals between spectra are 10, 30 and 60 s for 0…4, 4.5…10 and 10…16 min of the reaction, respectively. Inset: a time-course curve of the reaction

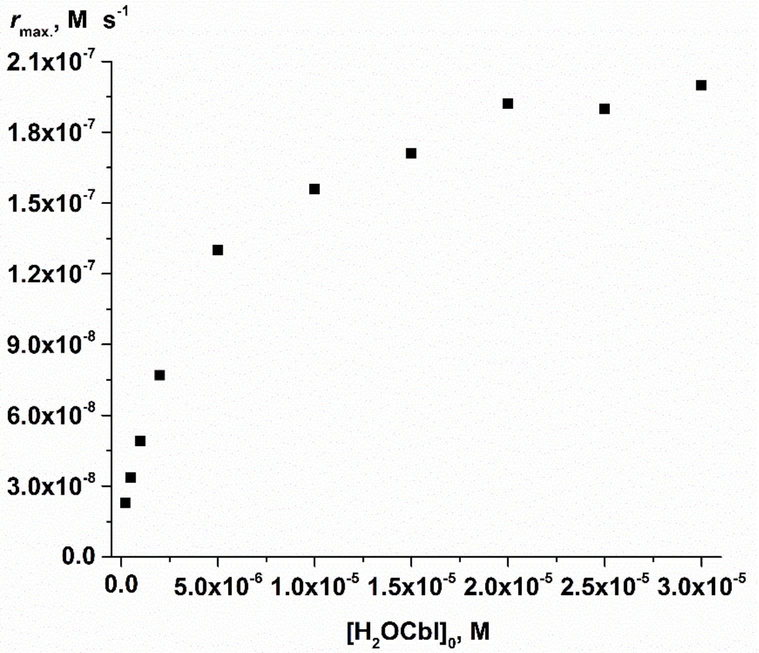

Figure S8. Plot of the maximum rate of the reaction between Orange II ($5.7\cdot10^{-5}$ M) and HSO_5^- ($5.0\cdot10^{-4}$ M) in the presence of H_2OCbl versus initial concentration of H_2OCbl at pH 7.4, 25.0 °C

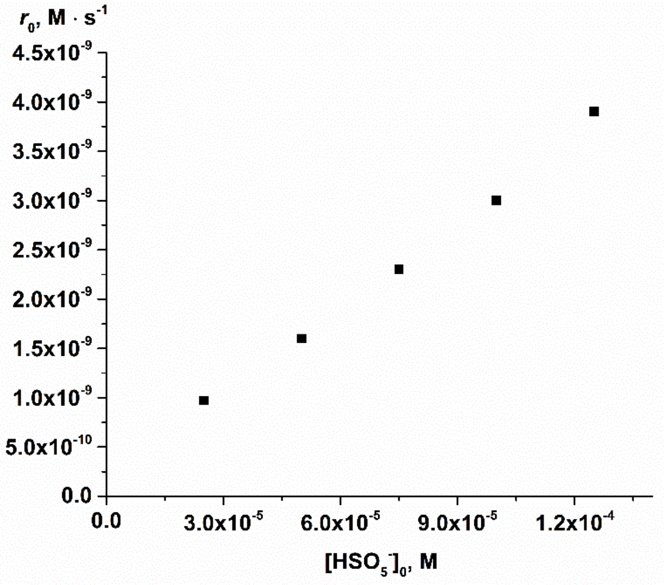

Figure S9. Plot of the initial rate of the reaction between Orange II ($5.7 \cdot 10^{-5}$ M) and HSO_5^- in the presence of H_2OCbl ($1.0 \cdot 10^{-6}$ M) versus initial concentration of HSO_5^- at pH 7.4, 25.0 °C

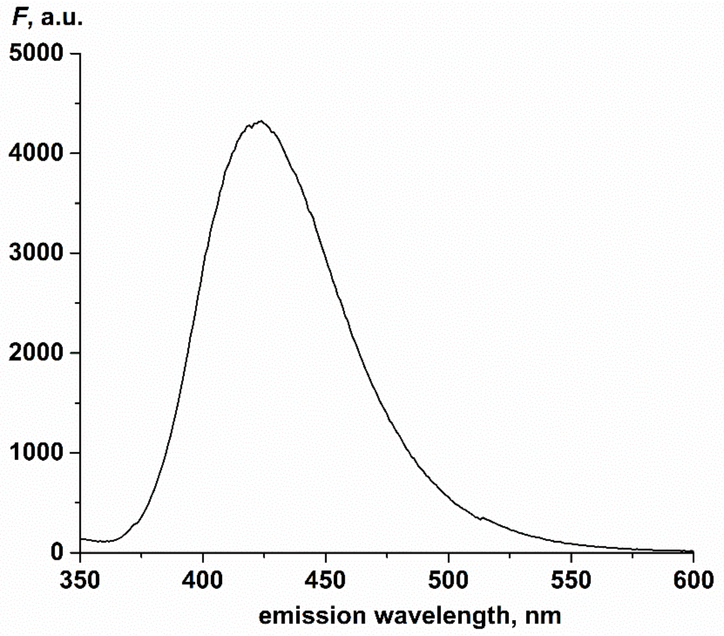

Figure S10. Fluorescence emission spectrum of 2-hydroxyterephthalic acid ($1.0 \cdot 10^{-6}$ M) at pH 7.4, 25.0 °C

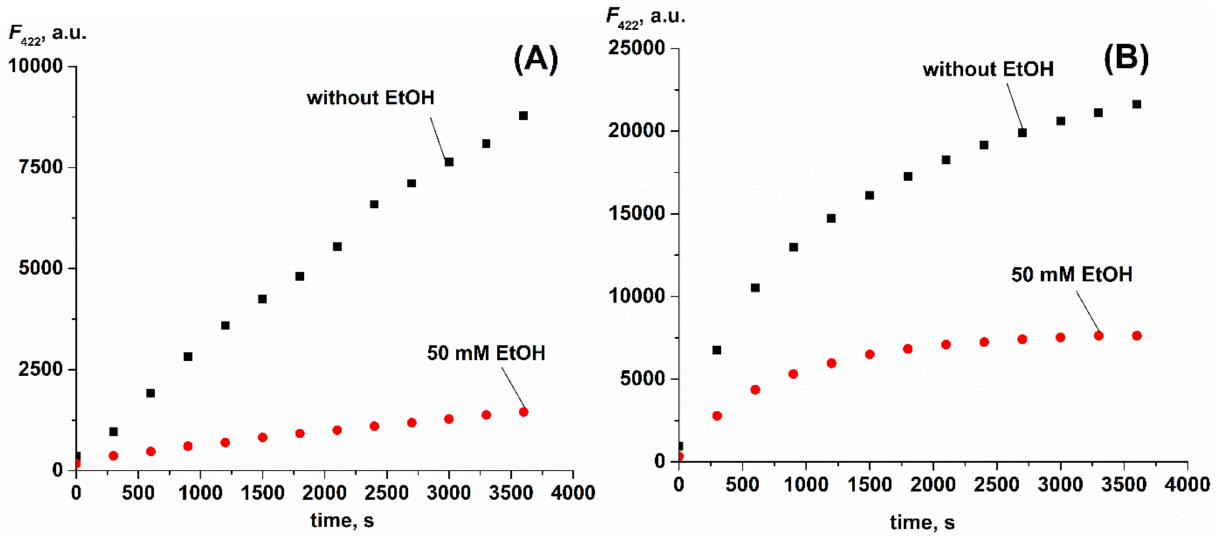

Figure S11. Plots of fluorescence intensity at 422 nm versus time for mixtures of terephthalic acid ($1.0 \cdot 10^{-3}$ M) with HSO_5^- ($5.0 \cdot 10^{-4}$ M; A) and with HSO_5^- ($5.0 \cdot 10^{-4}$ M) and H_2OCbl ($1.0 \cdot 10^{-6}$ M; B) in the absence and in the presence of ethanol (50 mM) at pH 7.4, 25.0 °C

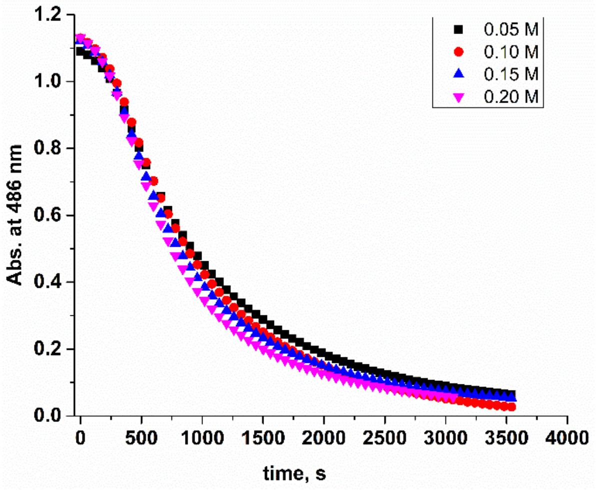

Figure S12. Time-course curves for the destruction of Orange II ($5.5 \cdot 10^{-5}$ M) by the mixture of H_2OCbl ($1.0 \cdot 10^{-6}$ M) with HSO_5^- ($5.0 \cdot 10^{-4}$ M) at pH 7.4, 25.0 °C in the presence of different phosphate buffer concentrations

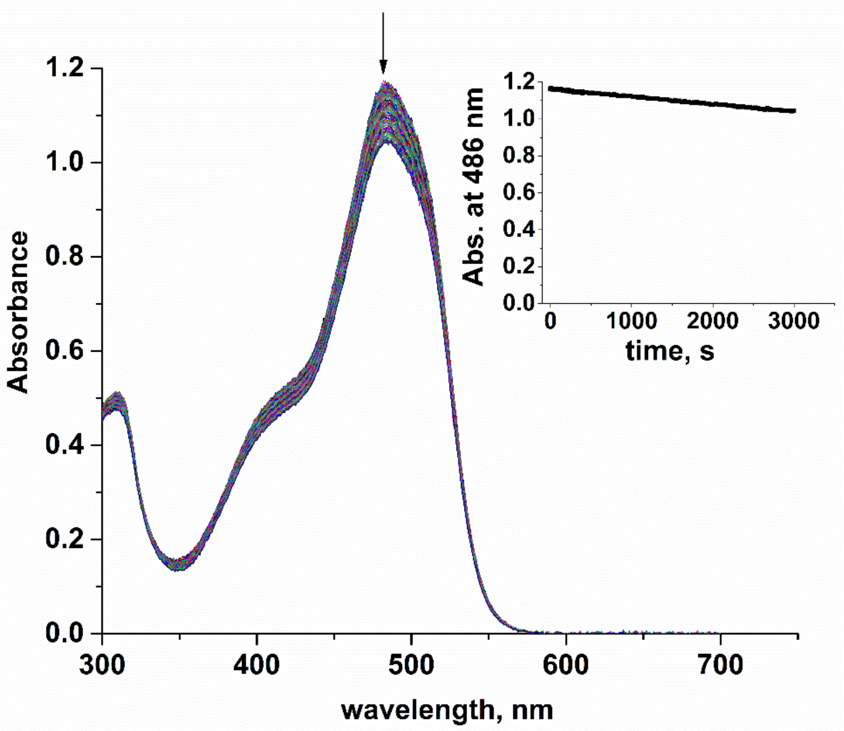

Figure S13. UV-vis spectra for the destruction of Orange II ($5.7\cdot10^{-5}$ M) by the mixture of H_2OCbl ($1.0\cdot10^{-6}$ M) with HSO_5^- ($5.0\cdot10^{-4}$ M) at pH 7.4, 25.0 °C in the presence of HCO_3^- ($5.0\cdot10^{-2}$ M). Time interval between spectra is 60 s. Inset: a time-course curve of the reaction

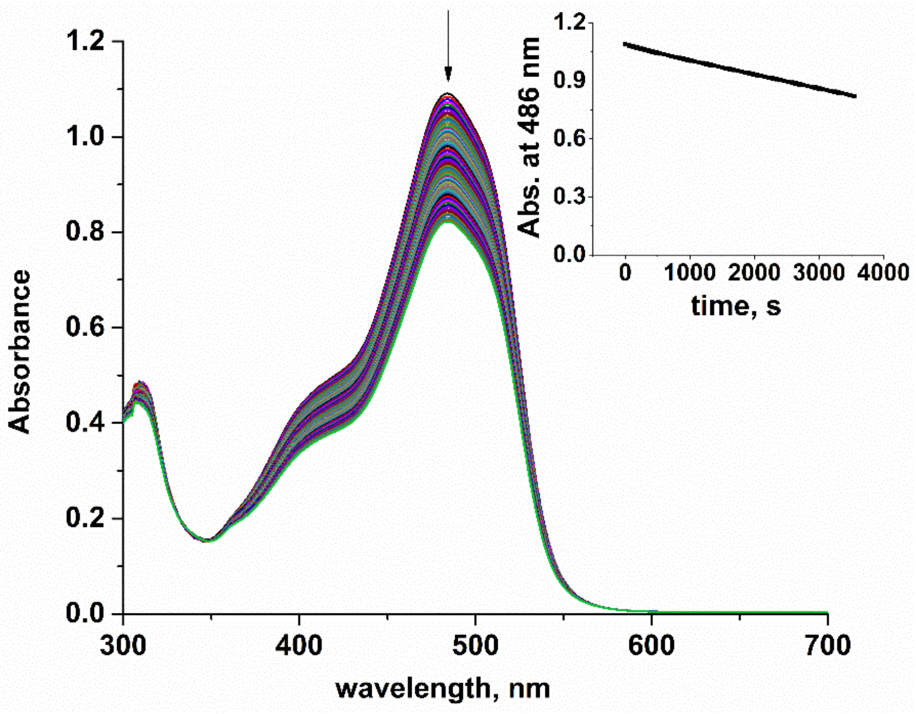

Figure S14. UV-vis spectra of the reaction between Orange II ($5.5\cdot10^{-5}$ M) and HSO_5^- ($5.0\cdot10^{-4}$ M) in the presence of CNCbl ($1.0\cdot10^{-6}$ M) at pH 7.4, 25.0 °C. Time interval between spectra is 60 s. Inset: a time-course curve of the reaction

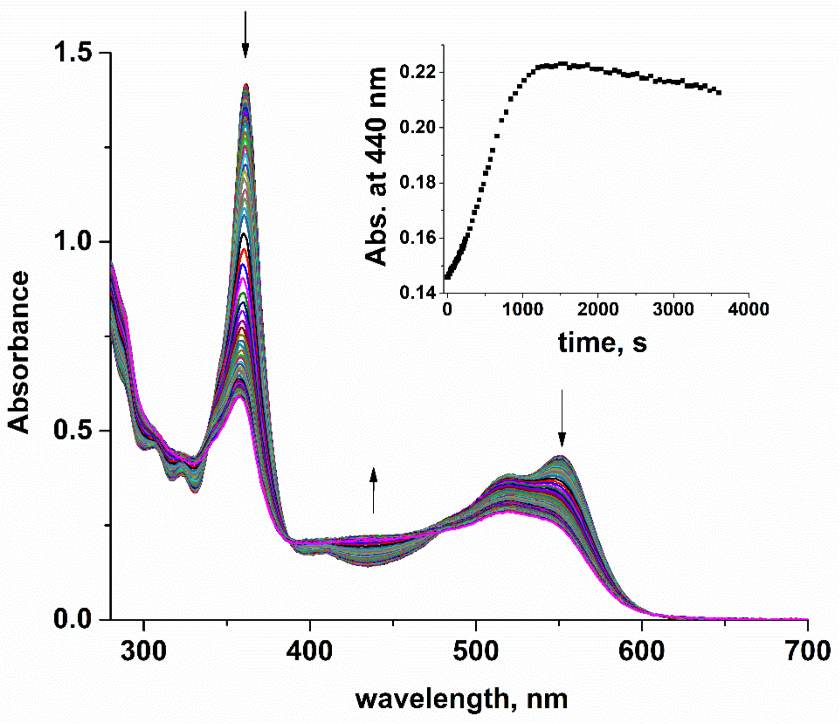

Figure S15. UV-vis spectra for the reaction between CNCbl ($5.0 \cdot 10^{-5}$ M) with HSO_5^- ($5.0 \cdot 10^{-4}$ M) at pH 7.0, 25.0 °C. Time interval between spectra is 60 s. Inset: a time-course curve of the reaction
